# The geometry of expertise

**DOI:** 10.3389/fpsyg.2014.00047

**Published:** 2014-02-04

**Authors:** María J. Leone, Diego Fernandez Slezak, Guillermo A. Cecchi, Mariano Sigman

**Affiliations:** ^1^Laboratorio de Neurociencia Integrativa, Departamento de Física, Facultad de Ciencias Exactas y Naturales, Universidad de Buenos AiresBuenos Aires, Argentina; ^2^IFIBA, CONICETBuenos Aires, Argentina; ^3^Departamento de Computación, Facultad de Ciencias Exactas y Naturales, Universidad de Buenos AiresBuenos Aires, Argentina; ^4^Computational Biology Center, T.J. Watson Research Center, International Business MachinesYorktown Heights, NY, USA; ^5^Universidad Torcuato Di TellaBuenos Aires, Argentina

**Keywords:** chess expertise, object representation, chunks, spatial proximity, attentional control

## Abstract

Theories of expertise based on the acquisition of chunk and templates suggest a differential geometric organization of perception between experts and novices. It is implied that expert representation is less anchored by spatial (Euclidean) proximity and may instead be dictated by the intrinsic relation in the structure and grammar of the specific domain of expertise. Here we set out to examine this hypothesis. We used the domain of chess which has been widely used as a tool to study human expertise. We reasoned that the movement of an opponent piece to a specific square constitutes an external cue and the reaction of the player to this “perturbation” should reveal his internal representation of proximity. We hypothesized that novice players will tend to respond by moving a piece in closer squares than experts. Similarly, but now in terms of object representations, we hypothesized weak players will more likely focus on a specific piece and hence produce sequence of actions repeating movements of the same piece. We capitalized on a large corpus of data obtained from internet chess servers. Results showed that, relative to experts, weaker players tend to (1) produce consecutive moves in proximal board locations, (2) move more often the same piece and (3) reduce the number of remaining pieces more rapidly, most likely to decrease cognitive load and mental effort. These three principles might reflect the effect of expertise on human actions in complex setups.

## Introduction

The focus of attention can be directed by exogenous (bottom-up) and endogenous (top-down) cues (Pylyshyn, 2007; Richard et al., 2008). The region of visual space to which attention is directed changes according to specific goals and tasks (Gilbert and Sigman, 2007; Vinckier et al., 2007). A classic example is Yarbus gaze experiment (where subjects had to view a complex image several times, each with a different instruction), he demonstrated that the sequence of eye fixations changed drastically according to the question the observer was trying to respond about the image (Yarbus, 1967; Tatler et al., 2010). Top-down control of attention can act over a wide range of categories, including location but also objects, goals, features, context, time … (Duncan, 1984; Chun and Jiang, 1998; Maunsell and Treue, 2006; New et al., 2007). The ability to direct attention to specific objects and categories changes with experience (Gilbert and Sigman, 2007) and, similarly, the ability to ignore salient cues requires inhibition mechanisms which are trainable (Cepeda et al., 1998).

Chess has been one of the most widely studied models of expertise (de Groot, 1978; Schultetus and Charness, 1999; Reingold et al., 2001a,b; Campitelli and Gobet, 2008; Connors et al., 2011; Bilalic et al., 2012). Chess experts recognize and recall chess positions accurately [chunk and templates theories (Chase and Simon, 1973; Gobet and Simon, 1996)], and develop heuristics that allow them to focus and explore only a few “good enough” moves (de Groot, 1978), substantially alleviating the search process.

As in other domains of perceptual expertise, chunk and templates acquisition theories reflect geometrical differences in the organization of perception between chess experts and novices: strong players recognize groups of pieces connected by functional relations as units (Gobet and Simon, 1996) and they also explore chess positions differently than novices [eye fixations are more centered in relationships between pieces (Reingold et al., 2001a)]. An implication of this theory is that expert representation is not anchored to the proximity between two pieces (Euclidean distance) and may instead be dictated by the intrinsic relation in the structure and grammar of the board. For instance, a bishop in one corner of the board which works in concert with a knight on the other side of the board to jointly attack an opponent square may be “functionally proximal” pieces in the mind of an expert, but “functionally distal” in the mind of a novice who does not recognize this relation. The same argument is true for other domains of expertise, for instance an expert soccer goalkeeper may bind together (spatially) distant properties of the field (where is the ball, where are the defenders and the attackers) which jointly may build an important cluster of features. Here we set out to examine explicitly this conjecture. We focus on chess which has three principal advantages to solve our goal: (1) The degree of proficiency can be quantified precisely with international systems of ratings (Elo, 1978), (2) The spatial layout in chess is well delimited by a square discrete grid of 64 locations, and (3) Capitalizing on chess internet servers we can base our conclusions on massive sets of data.

We reasoned that the movement of an opponent piece to a specific square constitutes an external attentional cue. The reaction of the player to this “perturbation” should reveal his internal representation of proximity. Specifically, we hypothesized that a naive player will tend to respond in proximal squares. Instead, we hypothesized that expert player responses are less likely to be governed by the spatial position of the opponent last move. Additionally, when this argument is expressed in terms of object oriented attention, we hypothesized that a novice player will more likely direct attention (concentrate) to a specific piece and hence produce repeated sequence of moves with the same piece. Instead, expert representation is directed to a more sophisticated pattern of pieces (chains of pawns, coordinated set of pieces working in concert…) and hence the sequence of moves should show fewer repetitions. If weak players focus more on individual pieces, we hypothesized they should tend to reduce the number of objects to be attended to avoid cognitive load. Finally, we examined some core aspects of chess strategy hypothesizing the experts would play more in-line with them than novices.

## Materials and methods

### Data acquisition

All games were downloaded from FICS (*Free Internet Chess Server*, http://www.freechess.org/), a free ICS-compatible server for playing chess games through Internet, with more than 300,000 registered users. This constitutes a quite unique experimental setup providing virtually infinite data (thousands of millions of moves).

Each registered user may be human or a computer player, and has associated a rating (Glicko rating, http://www.glicko.net/glicko.html) that indicates the chess skills strength of the player, represented by a number typically between 1000 and 3000 points. We defined two expertise levels: (a) strong players with rating higher than 1900 points and (b) weak players with rating between 1000 and 1400 points.

A regular game of chess contains about 40 moves from each player. A ply (plural plies) refers to one turn taken by a player. Hence, a chess game of 40 moves corresponds to 80 plies. We use the term “*next move*” to refer to two consecutive actions by the same player (white move 1: e4, white move 2: Nf3) and the term “next ply” to refer to consecutive actions by each player (ply 1: e4—white movement; ply 2: e5—black movement).

For each expertise group, we selected games from the FICS database (played from 2005 to 2013) with at least 80 plies, played between human players (of the same expertise group), with a total time budget of 180 s for each player and no increment. In order to make further comparisons, we generated 35 sets for each expertise group (each with 5000 games). These sets were built by date (i.e., set 1 contained mostly games from 2005 and set 35, from 2013). For each analysis we compared 35 values (one for each set) from the high rated expertise group with the 35 values from the low rated group.

In order to extend the results to longer time budgets, we replicated the analysis with games of 300 and 900 s per player. Because of the lower number of available games for these time budgets, we generated 35 datasets of 1500 games each (for each expertise group) for each of them. All other conditions were maintained.

### Movement distance measure

Piece location coordinates were organized in an 8*8 matrix (representing the 64 squares of the chess board). For each movement, we calculated two different measures (D_1_ and D_2_) to examine the proximity between successive actions in a game of chess. To do this, we define the initial square of a movement as the location of the moving piece before the movement and the final square of a movement as the location of the moving piece after this action.

D_1_ corresponds to the difference between the initial square of a piece movement and the final square of the previous movement by the opponent. In other words, this corresponds to the difference in location between two consecutive plies of the game. This is the observable which can be more easily mapped to classic attentional cues experiments. We make the analogy that the location where the opponent drops a piece is an attentional cue and observe the player's onset of his response relative to this cue.

D_2_ corresponds to the difference between the final squares of two successive moves from the same player. This is measured as the difference between the end-locations of ply(*n*+2) and ply(*n*).

For each measure we calculated the signed difference in squares in the x and y axis. Positive values of the y-axis indicate that the difference is shifted toward the upper side of the board. To do so we assumed the normal row conventions of chess (1 indicating white's first row and 8 black's first row). A positive difference in x-axis indicates a shift toward the left side and negative values a movement toward the right side of the board. Note that the differences in both axis take a range in the [−7, −6, …, 0, 1, …, 7] values. A value of zero indicates that there was not shift in that axis. For each measure (D_1_ and D_2_), we obtained a distance matrix for white moves and other for black moves, and we added them.

**Figure 1:**
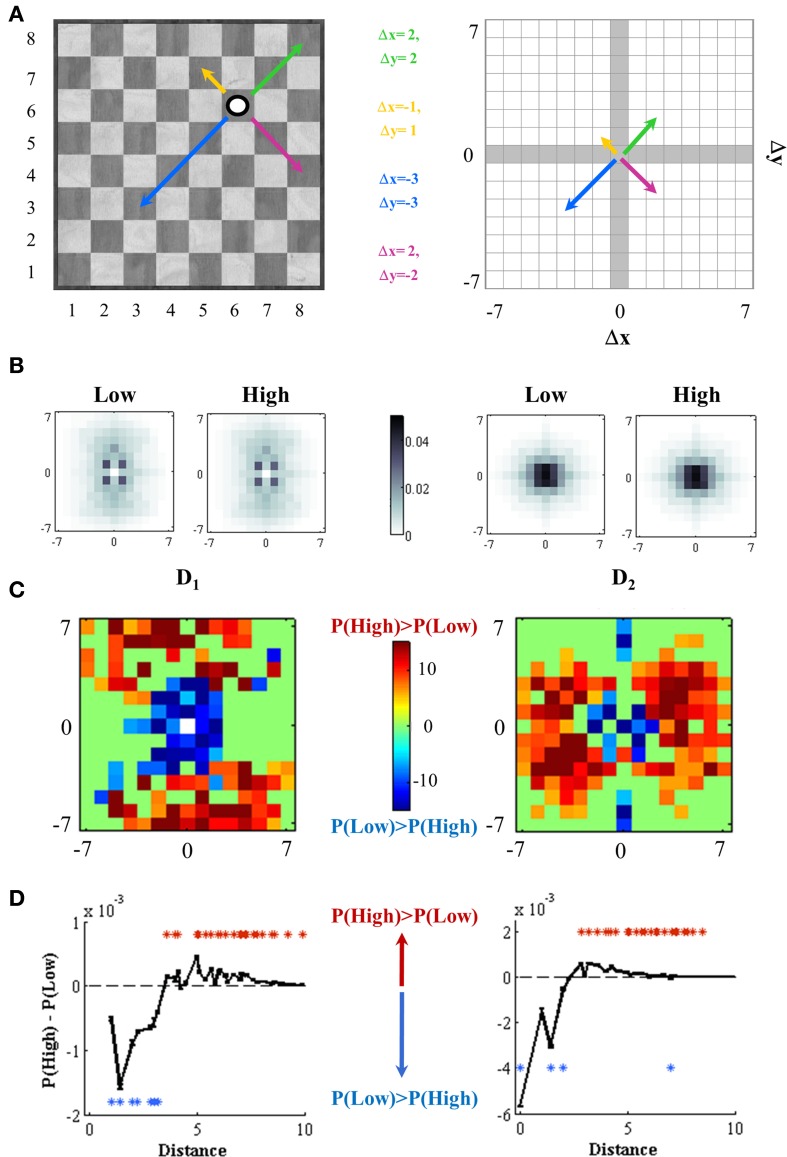
**Expertise level defines spatial effects on successive movements**. **(A)** Distance measurements. Distances between two squares over an 8*8 checkerboard can be measured subtracting the coordinates of one location (white circle) to the coordinates of other location (represented by the end of each arrow) on both x and y axis. The square where the white circle is located corresponds to *x* = 6 and *y* = 6, and four alternative locations are illustrated with color arrows. All possible distances between two squares of this 8 × 8 board are constrained to a 15*15 square, where now the coordinates of the white circle are (0, 0) and distance measures range from −7 to 7. The x axis shows the direction movement on number of columns (*x* < 0, left direction; *x* > 0, right direction) and the y axis represents movements on number of the rows (*y* < 0, down direction; *y* > 0, up direction). For example, the distance between the end of the yellow arrow (which is at *x* = 5 and *y* = 7 on the 8*8 board) and the white circle is calculated as the difference between the corresponding squares coordinates (Δ x = −1 and Δ y = 1). **(B)** Probability distributions of movement distances. We use two observables to assess locality effects on chess playing: D_1_ and D_2_ (see Methods). Probabilities to make a movement close to the previous one is higher at short distances, for both High and Low expertise levels. **(C)** Weak players made their movements closer to the previous one. We contrasted probability distributions for both High and Low rated players on each entry of the 15*15 distance square independently. *t*-value of each independent two-sample *t*-test (with *p*-value < 0.001, Bonferroni corrected for multiple comparisons) is color-coded. Positive (red) *t*-values indicate significantly higher probabilities for high rated players and negative (blue) values, for weaker players. **(D)** Radial or Euclidean distances. Distances were one-dimension collapsed and the difference between probabilities of making movements corresponding to a distance square [P_(High)_ – P_(Low)_] was plotted vs. each radial distance. High and low rated groups distributions were independently compared in each radial distance [two-sample *t*-test on each variable (D_1_ and D_2_)]. Red asterisks (*t* > 5.3 for D_1_ and *t* > 5.6 for D_2_) indicates distances were P_(High)_ is significantly higher than P_(Low)_; blue asterisks (*t* < −12.3 for D_1_ and *t* < −11 for D_2_), distances were P_(Low)_ > P_(High)_; in both *p* < 0.001, Bonferroni corrected for multiple comparisons. Dotted lines indicate distance values where the significances changes from weak to strong players.

For each independent set (35 per expertise group) we calculated the 15*15 matrices D_1mat_(*i,R*) and D_2mat_(*i,R*), where *i* ranges from 1 to 35 and *R* can be high or low rated group. Probabilities for each entry of the matrices were calculated as follow: (a)- for each movement (at least 40*5000 –number of moves*number of games-, white or black) we calculated the distance measure, (b) we counted the amount of movements which matched with each matrix entry, (c) we divided the number of movements on each entry by the total number of movements on the distance matrix. For each matrix, the entries indicate the probability to find successive plies (for D_1_) or moves (for D_2_) at the corresponding distance (see Figure 1) where indices 1 … 15, respectively, code distances [−7 … 7].

Our main experimental question is to investigate whether certain transitions (spatial differences between successive movements) differ between high and low rated players. To examine this we performed two-sample *t*-tests comparing high vs. low groups. We performed an independent *t*-test for each entry of the matrix (a total of 225 tests), each comparing the 35 values measured for the high and low expertise groups. From this analysis we generated a matrix oft-values which encodes the difference in probabilities for each entry between high and low rated players. Positive *t*-values indicate that this transition is more likely for high than for low rated players. *p*-values were corrected with a strict Bonferroni criterion for multiple comparisons, considering a difference as significant only if *p* < 0.001/225. For visualization purposes, *t*-values in comparisons that did not reach significance were set to *t* = 0.

We then collapsed the matrices D_1mat_(*i,R*) to their radial distance by the conventional formula *r* = (Δx^2^ + Δy^2^)^1/2^. This resulted on independent vectors of 34 dimensions for each *i* and *R*. Note that the possible differences in distance over the board is less than 15*15 since there is a lot of redundancy (for instance moving the king one square to the left, to the right, up or down, all correspond to a radial distance of 1). As before, we converted these distributions in a vector of *t*-values which encode the difference in probabilities for each index between high and low rated players. Positive *t*-values indicate that this transition is more likely for high than for low rated players. *p*-values were corrected with a strict Bonferroni criterion for multiple comparisons, considering a difference as significant only if *p* < 0.001/34.

### Piece repetition

This analysis is shown only for white moves (analyzing black moves yielded identical results). We encoded for each white move whether the piece moved was the same (1) or different (0) than the piece moved in the previous turn. The probability of moving the same piece twice depends on the number of pieces remaining of the board. Thus, we calculated the repetition probability as a function of the pieces remaining in the board independently for the 35 sets of each expertise level. This yielded vectors PR(*i,R,np*) where *i* indicates the group (1–35), *R* the expertise level (high or low) and *np* the number of pieces remaining on the board from 3 to 16 (there were not sufficient positions with one or two pieces remaining on the board when considering the first 40 moves). To quantify the main effects of expertise level and number of pieces on the repetition probability we made a Two-Way ANOVA test with number of pieces and expertise level as independent factors and their interactions. Then, we compared the distributions of high and low rating independently for each number of pieces with independent two-sample *t*-tests comparing high and low rated players. *p*-values were corrected with a strict Bonferroni criterion for multiple comparisons, considering a difference as significant only if *p* < 0.001/16.

### Number of pieces remaining on the board

This analysis is shown only for white moves (analyzing black moves yielded identical results). We calculated for each white move number (1–40) the number of white pieces remaining on the board (1–16). We then averaged this value for each move number (1–40) across all the games in each set. This yielded vectors NP(*i,R,n*) where *i* indicates the set (1–35), *R* the expertise level (high or low), and *n* the move number from 1 to 40. Note that this distribution as function of move number has to be monotonous decreasing. This would not be true in bughouse or crazy house variants of the games where a player can introduce a captured piece back to the board. We assessed the main effects of move number and expertise level with a Two-Way ANOVA test with move number and expertise level as independent factors and their interactions. Then, we compared the distributions of high and low rating independently for each move number with independent two-sample *t*-tests. *p*-values were corrected with a strict Bonferroni criterion for multiple comparisons, considering a difference as significant only if *p* < 0.001/40.

### Board distribution

This analysis is shown only for white moves (analyzing black moves yielded identical results). We calculated for white moves 5, 10, 20, 30, and 40 and for each piece the frequency of distribution along all squares of the board. Since the remaining amount of pieces between varies with expertise levels and the number of types of pieces is not the same (8 pawns, 2 bishops, one king,…) we normalized the occurrences by the average number of the piece type at each move number on each set. For example, the probability to have a knight on b1 in the move number 1 (in a set of an expertise level) is 0.98 and for g1 is 0.93, but at that moment there were 2 knights over the board (on average in that set), then the values for b1 and g1 were 0.49 and 0.465, respectively.

This results (for each piece type, move number, set and expertise level) in an 8*8 matrix which encodes the normalized average occupation of the corresponding piece along the board. Then, for each piece and move number, we compared the distributions along the board for high and low rating, with independent two-sample *t*-tests for each entry of the matrix. Positive *t*-values indicate that the occupation probability in a given entry is more likely for high than for low rated players. *p*-values were corrected with a strict Bonferroni criterion for multiple comparisons, considering a difference as significant only if *p* < 0.001/64. We reported results from knights, rooks and queen in the full matrix.

**Figure 2:**
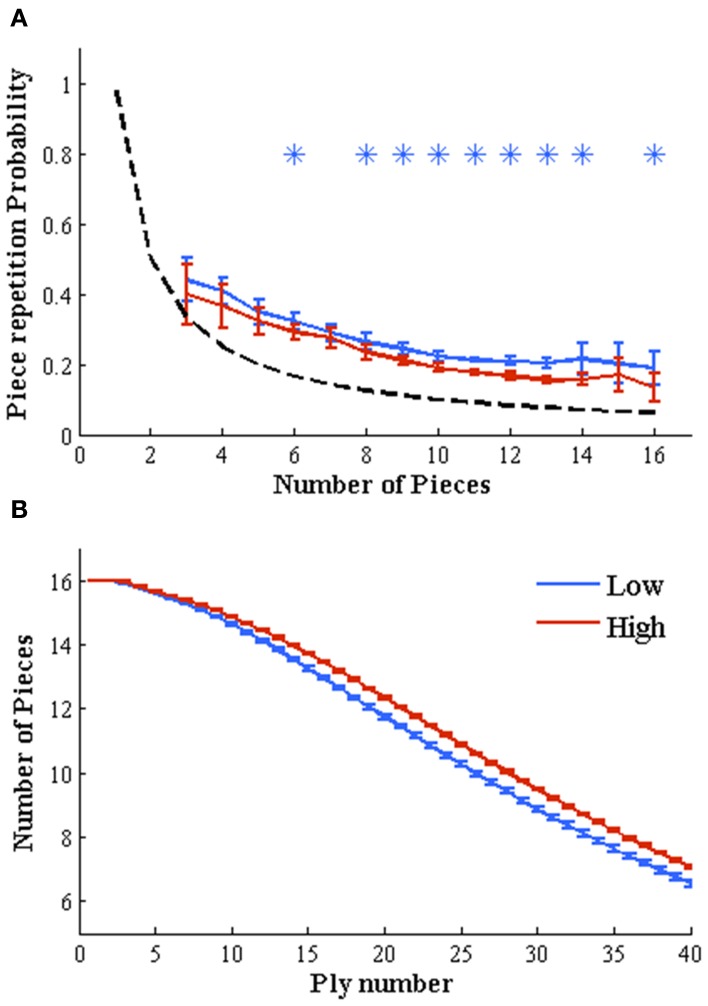
**Object-based mechanisms depend on expertise level**. **(A)** Weak players repeat the same piece on consecutive movements more frequently than strong players. Probabilities to repeat the same piece on two consecutive moves depends on the remaining number of pieces on the board. We plotted this probability vs. the number of pieces for both expertise levels. Independent two-sample *t*-tests (high vs. low expertise probabilities) for each number of pieces left (ranging from 1 to 16) evidence a higher probability to move the same piece on consecutive movements for low rated players, almost independently of the number of remaining pieces (*p < 0.001 Bonferroni corrected for multiple comparisons). Dashed black line shows the random threshold. **(B)** Low-rated players reduce the number of pieces more rapidly than high-rated players. The number of remaining pieces over the board, which change throughout the game (starting in 16 pieces for each player), is significantly higher for high rated players along the whole game (moves 3–40, independent two-sample *t*-tests on each move number, *p < 0.001 Bonferroni corrected for multiple comparisons), evidencing than low rated players exchange (or loss) their pieces more rapidly than stronger players.

Data is represented as mean ± *SD* (*n* = 35) for Figures 1C, 2A,B. Asterisks indicate significant differences at *p* < 0.001 (Bonferroni corrected). The same color code is maintained along the whole work: red indicates higher probabilities for strong players and blue for weaker players.

## Results

### Hypothesis 1

Low rated players make moves which are more proximal to their own last move and to the opponent precedent move.

D_1_ considers the difference between the initial location of a piece movement and the final location of the previous movement by the opponent. D_2_ corresponds to the difference between the final locations of two successive moves from the same player (see Materials and Methods and Figure 1A for a full description of how D_1_ and D_2_ are calculated).

For both expertise groups, we found a similar distribution of distances probabilities: all players tended to make their movements in squares close to the final location of their opponent last movement (D_1_) and to their own last movement (D_2_) (Figure 1B). However, and in accordance with our first hypothesis, lower rated players tend to make more movements in board locations which are proximal to the opponent's last movement (D_1_) and their own antecedent movement (D_2_). Instead, strong players showed higher densities of successive movements which were more scattered in space (Figure 1C)^1^. To quantify this observation we aggregated the distribution of spatial differences to a single scalar estimating the radial distance between the two successive movements (Figure 1D).

Results of D_1_ indicated that movements starting within a radius of 3.5 squares from the position where the opponent moved his piece were more likely in low rated players (*p* < 0.001 Bonferroni corrected) while movements outside of this radius where more likely for high rated players (*p* < 0.001 Bonferroni corrected). Similarly, results of D_2_ indicated that low rated players were more likely (*p* < 0.001 Bonferroni corrected) to make two consecutive moves with end-locations within a radius of 2.5 squares, while higher rated players made more likely moves beyond this radius.

Both of these results are extremely reliable through distances (Figure 1D). This indicates that while the specific geometry of the board may be subtle and specific to chess (the patterns of Figure 1C are complex) they organize on a synthetic rule by which low rated players tend to overplay more proximal and high rated players more distal moves.

Previous results were obtained using games with a short time budget (180 s). To avoid any confound related with the use of very short games, we repeated the exact analysis for games with longer time budgets (300 and 900 s per player) and we found the same results (Figures S1, S2).

### Hypothesis 2

Low rated players are more likely to move the same piece in consecutive turns.

The previous results suggest that low rated players have a narrower (or more focal) spatial window of attention. Attention can also be directed to objects (Richard et al., 2008). We examine the hypothesis that throughout a game, low rated players are more focused in a specific piece than high rated players, who may drive attention to schemas assembling sets of pieces (pawn chains, several pieces converging in a square or a plan …). To this aim, we simply measured the probability of repeating a piece in two consecutive moves. A repetition is counted only when the exact same piece (not the same type of piece) is moved twice. If a player moves a pawn and in the next turn moves another pawn, this is considered as a different piece movement.

The probability of moving the same piece twice depends on the number of pieces remaining of the board. Thus, we calculated the repetition probability as a function of the pieces remaining in the board (Figure 2A). The repetition probability decreased with the number of pieces for both groups but remained above chance levels. This is expected since: (1) some pieces actually cannot move and (2) players rarely consider all pieces in the board as candidates to move. As we had hypothesized, lower rated players produced more repetitions reflecting that attention (or their strategies or conception of plans) is more likely to be constrained to a single piece. To quantify this observation we first submitted the data to a Two-Way ANOVA test with number of pieces and expertise level as independent factors and their interactions. Results showed a main effect of both factors (Expertise, *p* < 0.0001, *F* = 91.8, *df* = 1; Number of Pieces: *p* < 0.0001, *F* = 181.6, *df* = 15 and Interaction, *p* < 0.0001, *F* = 7.5, *df* = 15). We followed this test with independent two sample *t*-tests (corrected with a strict Bonferroni criterion for multiple comparisons) for each number of pieces left, comparing the distributions for high and low rated players. Each value of the distribution is obtained from one of the 35 different sets of each expertise level. All comparisons consistently showed greater repetition probability for lower than higher rated player. This effect was significant when 6, 8–14, and 16 pieces remaining on the board [*t*_(34)_ < −5.2, *p* < 0.001].

As for the first hypothesis, we repeated the piece repetition analysis using games with longer time budgets (Figures S3A,B) and we replicated the results found for 180 s games.

### Hypothesis 3

Since weak players focus more on individual pieces it is expected that it is effortful for them to work on boards with many pieces. We expect that weaker players will tend to simplify the position to avoid mental effort (Koechlin and Summerfield, 2007).

We compared the amount of pieces as a function of move number for both expertise levels. As expected the number of pieces started in 16 (initial configuration) and smoothly decreased to an average of about 7 pieces by move 40. Beyond this main trend we observed that after the first five moves, the distributions of remaining pieces for high and low rated players bifurcate. This analysis shows that, as we had hypothesized, weaker players exchange pieces more rapidly than stronger players. To quantify this observation we first made a Two-Way ANOVA test with number of pieces and expertise level as independent factors and their interactions. Results showed a main effect of both factors (Expertise, *p* < 0.0001, *F* = 4.5*10^4^, *df* = 1; Move number: *p* < 0.0001, *F* = 2.2*10^5^, *df* = 39 and Interaction, *p* < 0.0001, *F* = 296, *df* = 39). We followed this test with independent two-sample *t*-tests (corrected with a strict Bonferroni criterion for multiple comparisons) for each move number, comparing the distributions for high and low rated players. All comparisons consistently showed significant greater number of pieces along the whole game (move numbers 3–40) for high rated players [*t*_(34)_ > 15 for move numbers 4–6, *t*_(34)_ > 27 for move numbers 3 and 7–40, *p* < 0.001]. Once again, we repeated the previous analysis for 300 and 900 s games (Figures S3C,D) replicating the results found for 180 s time budget.

The results described above show that weaker players tend to produce consecutive moves in proximal board locations, more often moving the same piece and exchanging pieces more rapidly to reduce the number of remaining pieces. These three principles reflect consistent general findings which might reflect the effect of expertise on human actions in complex setups.

Beyond these three principles there are idiosyncratic aspects of the game of chess which relate to piece value, with the way the move on the board and where they are more effective, which dictate a specific strategy in the board. Expert play is expected to be more in-line with certain strategic themes. Here we examined three core aspects of chess strategy: (1) Knights are more effective when they are centralized, (2) Rooks play first along the 1st row to find an optimal centralized column where they are effective, (3) The queen should not risk going for long travels early in the game.

**Figure 3:**
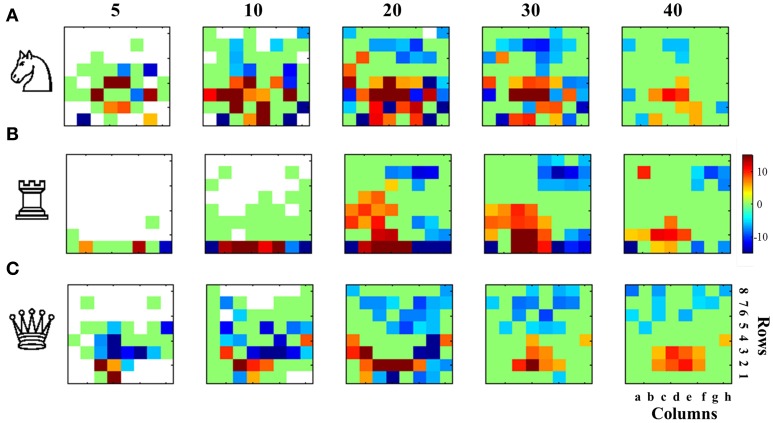
**Piece distribution over the board reveals domain-specific and expertise-dependent strategies**. Probabilities to find a type of piece (normalized by the number of remaining pieces of this type) in each square (8*8 checkerboard) were compared for high and low expertise groups at different game stages (moves 5, 10, 20, 30, and 40). The *t*-value resulting of the two-sample *t*-test (high vs. low expertise group), in each square is color coded for those significantly different comparisons with *p* < 0.001 Bonferroni corrected for multiple comparisons. Red positive values indicate significantly higher probabilities for strong players and blue negative values, significantly higher probabilities for weaker players. **(A)** Knights, **(B)** Rooks, and **(C)** Queen occupancy comparisons reveal that strong players centralize more their knights and rooks, delaying the queen development, compared with lower rated players delaying more the development of knights and rooks (but not the development of the queen, which is almost always not good) and/or occupying more advanced squares with all. It should be noted that the this figure represents the differential occupancy of each square, showing that strong or weak players locate each type of piece comparatively more frequently than the other group.

At different stages of the game (moves 5, 10, 20, 30, and 40) we calculated the average density of white pieces (N: knights, R: Rooks, Q: Queen) along the board. For each square we performed a two-sample *t*-test comparing the distribution of densities for the high and low rated players and corrected for multiple comparisons with a strict Bonferroni criterion. Results showed that, as expected, knights were significantly more centralized (over the whole board) for higher rated players and were more likely to be found in their initial square (b1 or g1) or advanced in the enemy camp for weaker players (Figure 3A). Rook movement revealed that by move 5 higher rated players are more likely to have castled and by move 10 a higher probability of centralizing the rooks on the 1st row (Figure 3B). Rooks position in weaker players instead was much more likely to be in the initial squares (a1 and h1). Also, as with knights, weaker players are more likely to advance the rook in the enemy camp. Another consistent finding is that stronger players place their rooks in the queen-side (left side of the board for white). Instead weaker players more rapidly attack on the king-side: for instance by move 30, white rooks are more likely to be found in squares close to the opponent-king location. This reflects a more positional play and a less direct tendency of directly going to mate the enemy king for high rated players. Finally, as expected, stronger players tend to postpone queen development (by move 5 and 10 the queen is more likely to be in the initial square d1) and throughout the game develop the queen on the first rows (Figure 3C). We emphasize that these results do not convey information about the absolute distribution of occupation of a piece. Instead they reflect differential distributions, i.e., indicating whether a given pieces is more likely to be occupied by stronger or weaker players. To avoid confusion Figure S4 shows the average degree of occupancy of these pieces throughout the game for each expertise level. As for previous hypotheses, we replicated and found similar results for games with longer time budgets (see Figure S5).

## Discussion

Here we showed differences in general and domain-specific patterns of actions depending on expertise level. We found that weaker players play more locally, tend to focus sequences of actions in the same piece and more rapidly exchange pieces to reduce the total number of pieces on the board. Our working hypothesis is that these observations reflect a different focus of attention to space and objects with expertise.

Our first working hypothesis was that the end location of a movement (the place where a piece is located) functions like a spatial cue in the board space. Our conjecture is that the tendency to continue playing close to that cue is a reflection of the persistence of attention to this location.

Attention involves a sequence of operations: 1- attentional shifting to the target location, 2-attentional engagement, 3-attentional disengagement (Posner and Petersen, 1990; Fox et al., 2002; Koster et al., 2006). The persistence of play in a given location has three likely and related explanations: a first possibility is that novices have difficulties disengaging attention from the cued place in the last movement (Sheridan and Reingold, 2013). Another possibility is that expert players have more effective preattentive mechanisms to encode saliency mechanisms in peripheral locations of the board (Sigman and Gilbert, 2000; Intriligator and Cavanagh, 2001; Pylyshyn, 2007; Vinckier et al., 2007). Chess masters have an advantage for the recognition of chess pieces (Saariluoma, 1995; Kiesel et al., 2009; Bilalic et al., 2010) and chess themes (Reingold et al., 2001a,b), which may serve as salient detector providing new cues which compete with the previous spatial cue. A third possibility is that expert players can attend to themes or schemas (strings of pieces) and the focus of attention is spread over the whole attended object (Houtkamp et al., 2003; Alvarez and Scholl, 2005; Richard et al., 2008). In line with this idea, the size and form of the selection window has been proposed to be controlled by top-down mechanisms and dependent on the task difficulty (Belopolsky and Theeuwes, 2010).

Our results based on the spontaneous distributions of actions during a game are consistent with a recent report by Sheridan and Reingold analyzing the distribution of attention in the Einstellung Effect (Sheridan and Reingold, 2013). The authors present a problem in which there is a move which almost indefectibly attracts attention (for instance a region on the board where there seems to be mate, with the king exposed and many pieces attacking it). In this construction, the best move is away from this specific location of the board. Weaker players very often do not consider this optimal (but distant from a very salient location) move and their gaze remains in the Einstellung region of the board. Instead, stronger players can disengage from this location which allows them to find the distant and optimal move (Sheridan and Reingold, 2013).

Weaker players are less likely to organize the representation of the board in large chunks (Chase and Simon, 1973; Gobet and Simon, 1996) and thus, comparing variations when many pieces remain on the board requires more attentional shifts and executive function. Koechlin and colleagues have coined the idea of a “lazy” executive system which is triggered only when strictly needed (Koechlin and Summerfield, 2007). Combining these premises, we reasoned that all players will seek to minimize effort. To achieve this, weaker players will prefer positions with less number of pieces on the board. As expected by this prediction we show that weak players tend to “simplify” the problem by more rapidly removing pieces of the board consistently.

We initially chose using 180 s games to test our hypotheses based on: 1- evidences showing that rapid processes (related with pattern recognition) rather than slow processes (fundamentally, search mechanisms) are responsible of chess expertise (Burns, 2004; Sigman et al., 2010), 2- the time used for each movement is close-related with the common psychological experiments and 3- the FICS database for 180 s games is larger than those for longer time budgets. However, we also replicated all our results for longer time budgets (300 and 900 s) indicating the robustness of the conclusions and absence of 180 s time budget-related artifacts.

The effect size of all our results is small but consistent with our working hypotheses and each result was replicated in three different time budgets, favoring the consistency and reliability of our results.

Our study focused on chess but there is no reason to think that the main of the conclusions derived here (with the exception of specific Strategic patterns shown in Figure 3) are specific to chess and hence are likely be generalized to the effect of expertise on other domains of human action and decision making (i.e., novice car drivers focusing his/her attention only in the front road but not in the car mirrors or novice sport players deciding their actions based only in the ball location because they are not able to simultaneously attend to their partners and opponents locations).

### Conflict of interest statement

The authors declare that the research was conducted in the absence of any commercial or financial relationships that could be construed as a potential conflict of interest.

## Footnotes

^1^Note that D_2_ has a non-zero probability in the origin which corresponds to two consecutive moves by one player which have the same end-location. This can only happen when a player captures twice in the same location. Instead D_1_ has a strict zero probability in the origin since a player cannot make a move starting in the square where the opponent has moved a piece.

## Supplementary material

Figure S1 **Spatial effects are also observed in longer games: 300 s time budget.** **(A)** Probability distributions of movement distances. As for 180 s games (Figure 1B), probabilities to make a movement close to the previous one are higher at short distances, for both expertise levels.** (B)** Weak players made their movements closer to the previous one. The same analysis explained for 180 s games was made for 300 s games, obtaining similar results (see Figure 1C). Probability distributions for both High and Low rated players were contrasted on each entry of the 15*15 distance square independently. *t*-value of each independent two-sample *t*-test (with *p*-value < 0.001, Bonferroni corrected for multiple comparisons) is color-coded. Positive (red) *t*-values indicate significantly higher probabilities for high rated players and negative (blue) values, for weaker players. **(C)** Radial or Euclidean distances. Again, as in Figure 1D for 180 s games, distances were one-dimension collapsed and the difference between probabilities of making movements corresponding to a distance square [P_(High)_ – P_(Low)_] was plotted vs. each radial distance. High and low rated groups distributions were independently compared in each radial distance [two-sample *t*-test on each variable (D_1_ and D_2_)]. Red asterisks (*t* > 5.2 for D_1_ and *t* > 4.9 for D_2_) indicates distances were P_(*High*)_ is significantly higher than P_(*Low*)_; blue asterisks (*t* < −7.3 for D_1_ and *t* < −7.8 for D_2_), distances were P_(Low)_ > P_(High)_; in both *p* < 0.001, Bonferroni corrected for multiple comparisons.

Figure S2 **Spatial effects are also observed in longer games: 900 s time budget.** **(A)** Probability distributions of movement distances. As for 180 s (Figure 1B) and 300 s (Figure S1A) games, probabilities to make a movement close to the previous one are higher at short distances (for both expertise levels). **(B)** Weak players made their movements closer to the previous one. The same analysis explained for 180 s and 300 s games was made for 900 s games, obtaining similar results (see Figure 1C). Probability distributions for both high and low rated players were contrasted on each entry of the 15*15 distance square independently. *t*-value of each independent two-sample *t*-test (with *p*-value < 0.001, Bonferroni corrected for multiple comparisons) is color-coded. Positive (red) *t*-values indicate significantly higher probabilities for high rated players and negative (blue) values, for weaker players. **(C)** Radial or Euclidean distances. Again, as for 180 s games (Figure 1D) and 300 s games (Figure S1C), distances were one-dimension collapsed and the difference between probabilities of making movements corresponding to a distance square [P_(High)_ – P_(Low)_] was plotted vs. each radial distance. High and low rated groups distributions were independently compared in each radial distance [two-sample *t*-test on each variable (D_1_ and D_2_)]. Red asterisks (*t* > 6.9 for D_1_ and *t* > 4.9 for D_2_) indicates distances were P_(High)_ is significantly higher than P_(Low)_; blue asterisks (*t* < −5.3 for D_1_ and *t* < −5 for D_2_), distances were P_(Low)_ > P_(High)_; in both *p* < 0.001, Bonferroni corrected for multiple comparisons.

Figure S3 **For longer games (300 and 900 s time budget), object-based mechanisms also depend on expertise level.** **(A,B)** Weak players repeat the same piece on consecutive movements more frequently than strong players. As for 180 s games (Figure 2A), we plotted the probability to repeat the same piece on successive movements vs. the number of pieces for both expertise levels for 300 and 900 s games. To quantify these observations we first submitted the data to a Two-Way ANOVA test with number of pieces and expertise level as independent factors and their interactions. Results for 300 s games showed a main effect of both factors (Expertise, *p* < 0.0001, *F* = 41.9, *df* = 1; Number of Pieces: *p* < 0.0001, *F* = 61.6, *df* = 15 and Interaction, *p* < 0.0001, *F* = 10.1, *df* = 15). Results for 900 s games showed a main effect of both factors (Expertise, *p* < 0.0001, *F* = 197.8, *df* = 1; Number of Pieces: *p* < 0.0001, *F* = 89.7, *df* = 15 and Interaction, *p* < 0.0001, *F* = 31.4, *df* = 15). We followed these tests with independent two sample *t*-tests (corrected with a strict Bonferroni criterion for multiple comparisons) for each number of pieces left, comparing the distributions for high and low rated players. Each value of the distribution is obtained from one of the 35 different sets of each expertise level. All comparisons consistently showed greater repetition probability for lower than higher rated player. For 300 s games, this effect was significant for 7–14 pieces remaining on the board [*t*_(34)_ < −4.9, *p* < 0.001]. For 900 s games, this effect was significant for 8–16 pieces remaining on the board [*t*_(34)_ < −6.4, *p* < 0.001]. Dashed black line shows the random threshold. **(C,D)** Low-rated players reduce the number of pieces more rapidly than high-rated players. As it was previously showed for 180 s games, the number of remaining pieces over the board is significantly higher for high rated players almost for the whole game for both 300 and 900 s games. First, we made a two-way ANOVA test with number of pieces and expertise level as independent factors and their interactions showed a main effect of both factors. ANOVA results for 300 s games: Expertise, *p* < 0.0001, *F* = 1.3*10^4^, *df* = 1; Move number: *p* < 0.0001, *F* = 3.2*10^4^, *df* = 39 and Interaction, *p* < 0.0001, *F* = 175, *df* = 39. ANOVA results for 900 s games: Expertise, *p* < 0.0001, *F* = 0.98*10^4^, *df* = 1; Move number: *p* < 0.0001, *F* = 2.2*10^4^, *df* = 39 and Interaction, *p* < 0.0001, *F* = 79, *df* = 39. We followed these tests with independent two-sample *t*-tests (corrected with a strict Bonferroni criterion for multiple comparisons) for each move number, comparing the distributions for high and low rated players. All comparisons consistently showed significant greater number of pieces almost along the whole game [in 300 s games, move numbers 3, and 8–40, *t*_(34)_ > 5, *p* < 0.001; in 900 s games, move numbers 3 and 7–40, *t*_(34)_ > 6, *p* < 0.001] for high rated players, evidencing than low rated players exchange (or loss) their pieces more rapidly than stronger players in 300 and 900 s games.

Figure S4 **Occupation distribution of pieces throughout the game for both expertise levels.** Probabilities to find a **(A)** Knight, a **(B)** Rook, or the **(C)** Queen at each square of the chess board is represented for each rating group at different game stages (move numbers 5, 10, 20, 30, and 40). It should be noted that both groups occupy almost the same squares along the board (there are not “exclusive” squares), but some places are comparatively more occupied by weak or strong players (see Figure 3).

Figure S5 **Piece distribution over the board also reveals domain-specific and expertise-dependent strategies in longer games (300 and 900 s time budget).** Probabilities to find a type of piece (normalized by the number of remaining pieces of this type) in each square (8*8 checkerboard) were compared for high and low expertise groups at different game stages (moves 5, 10, 20, 30, and 40). The *t*-value resulting of the two-sample *t*-test (high vs. low expertise group), in each square is color coded for those significantly different comparisons with *p* < 0.001 Bonferroni corrected for multiple comparisons. Red positive values indicate significantly higher probabilities for strong players and blue negative values, significantly higher probabilities for weaker players. **(A,D)** Knights, **(B,E)** Rooks, and **(C,F)** Queen occupancy comparisons reveal that strong players centralize more their knights and rooks, delaying the queen development, compared with lower rated players delaying more the development of knights and rooks (but not the development of the queen, which is almost always not good) and/or occupying more advanced squares with all. It should be noted that the this figure represents the differential occupancy of each square, showing that strong or weak players locate each type of piece comparatively more frequently than the other group. **(A–C)** correspond to 300 s games. **(D–F)** correspond to 900 s games.
