# Supplementary figures and images for: The geometry of expertise
Source: Front Psychol. 2014 Feb 4;5:47. doi: 10.3389/fpsyg.2014.00047 (PMC3913042; doi:10.3389/fpsyg.2014.00047)

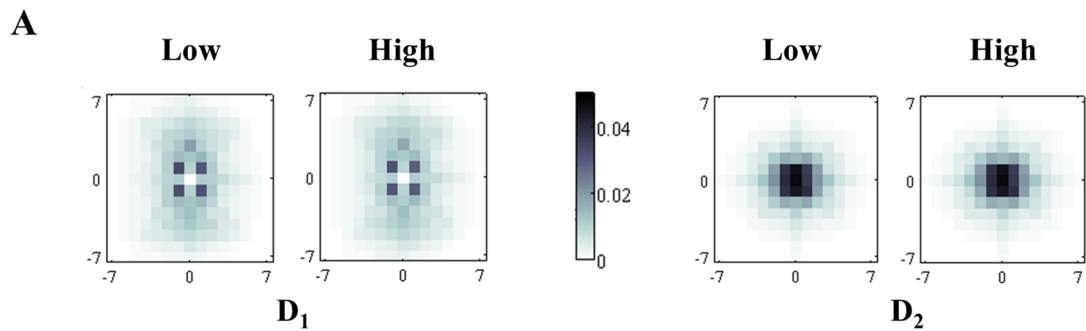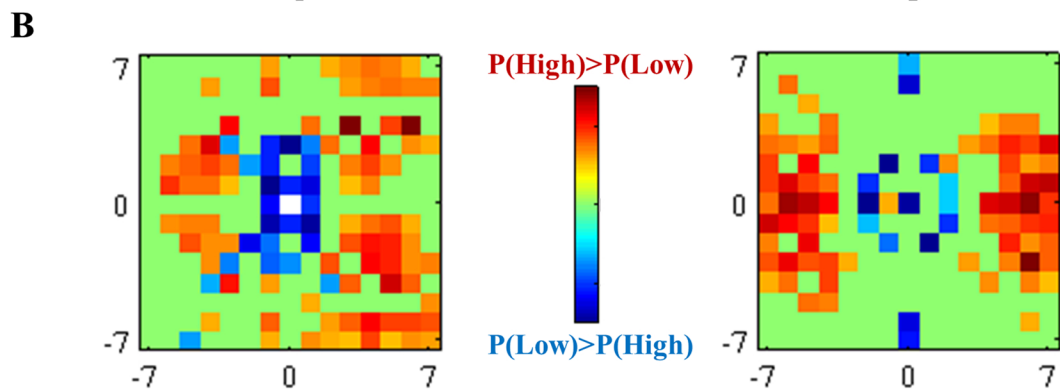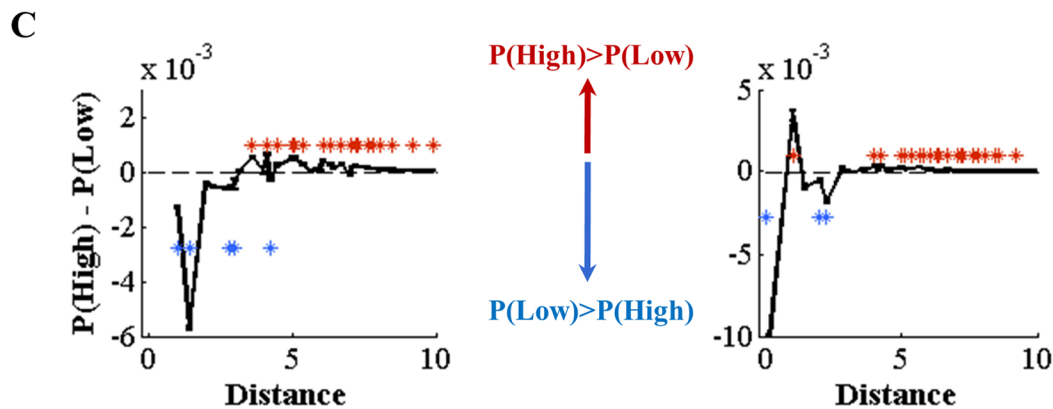

Supplement: Figure S1 — Spatial effects are also observed in longer games: 300 s time budget. (A) Probability distributions of movement distances. As for 180 s games (Figure 1B), probabilities to make a movement close to the previous one are higher at short distances, for both expertise levels. (B) Weak players made their movements closer to the previous one. The same analysis explained for 180 s games was made for 300 s games, obtaining similar results (see Figure 1C). Probability distributions for both High and Low rated players were contrasted on each entry of the 15*15 distance square independently. t-value of each independent two-sample t-test (with p-value < 0.001, Bonferroni corrected for multiple comparisons) is color-coded. Positive (red) t-values indicate significantly higher probabilities for high rated players and negative (blue) values, for weaker players. (C) Radial or Euclidean distances. Again, as in Figure 1D for 180 s games, distances were one-dimension collapsed and the difference between probabilities of making movements corresponding to a distance square [P(High) – P(Low)] was plotted vs. each radial distance. High and low rated groups distributions were independently compared in each radial distance [two-sample t-test on each variable (D1 and D2)]. Red asterisks (t > 5.2 for D1 and t > 4.9 for D2) indicates distances were P(High) is significantly higher than P(Low); blue asterisks (t < −7.3 for D1 and t < −7.8 for D2), distances were P(Low) > P(High); in both p < 0.001, Bonferroni corrected for multiple comparisons. [file Presentation1.PDF]

A

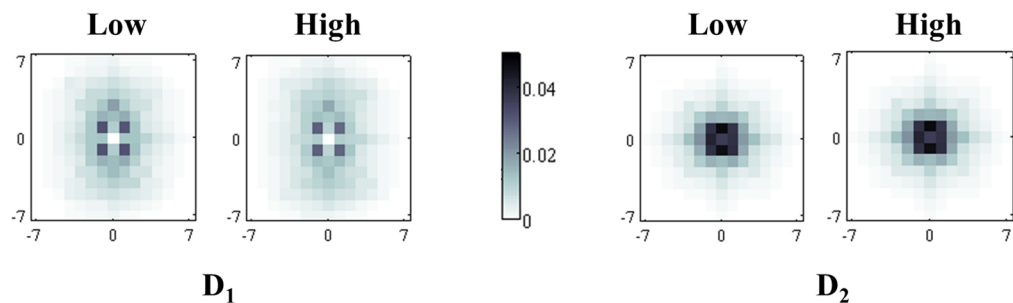

B

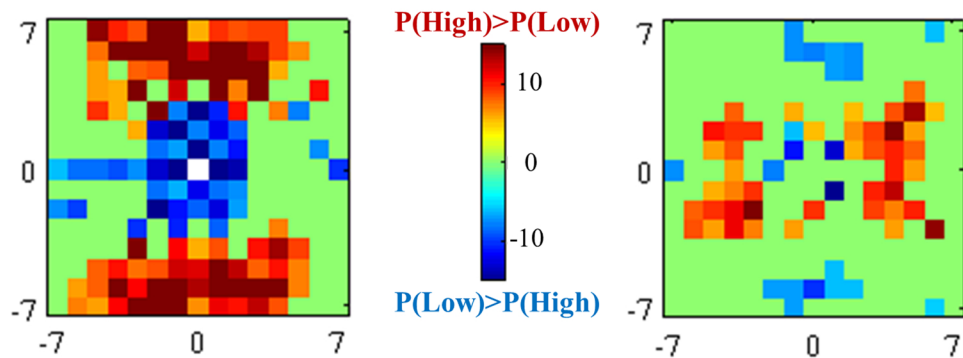

C

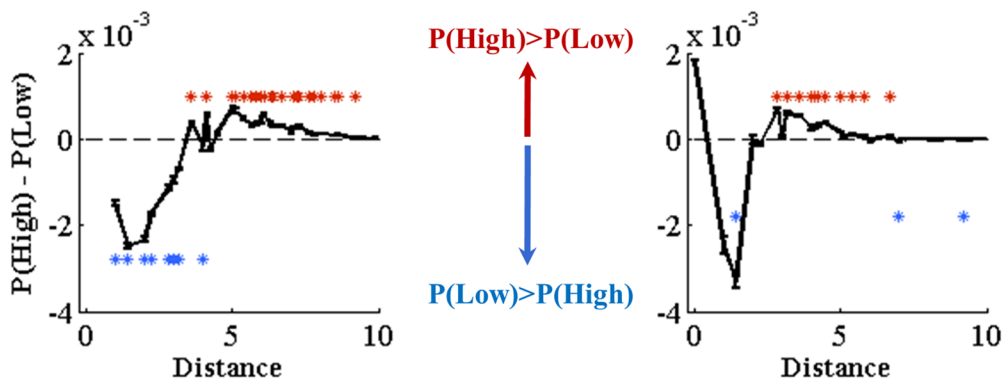

Supplement: Figure S2 — Spatial effects are also observed in longer games: 900 s time budget. (A) Probability distributions of movement distances. As for 180 s (Figure 1B) and 300 s (Figure S1A) games, probabilities to make a movement close to the previous one are higher at short distances (for both expertise levels). (B) Weak players made their movements closer to the previous one. The same analysis explained for 180 s and 300 s games was made for 900 s games, obtaining similar results (see Figure 1C). Probability distributions for both high and low rated players were contrasted on each entry of the 15*15 distance square independently. t-value of each independent two-sample t-test (with p-value < 0.001, Bonferroni corrected for multiple comparisons) is color-coded. Positive (red) t-values indicate significantly higher probabilities for high rated players and negative (blue) values, for weaker players. (C) Radial or Euclidean distances. Again, as for 180 s games (Figure 1D) and 300 s games (Figure S1C), distances were one-dimension collapsed and the difference between probabilities of making movements corresponding to a distance square [P(High) – P(Low)] was plotted vs. each radial distance. High and low rated groups distributions were independently compared in each radial distance [two-sample t-test on each variable (D1 and D2)]. Red asterisks (t > 6.9 for D1 and t > 4.9 for D2) indicates distances were P(High) is significantly higher than P(Low); blue asterisks (t < −5.3 for D1 and t < −5 for D2), distances were P(Low) > P(High); in both p < 0.001, Bonferroni corrected for multiple comparisons. [file Presentation2.PDF]

**A****300 s**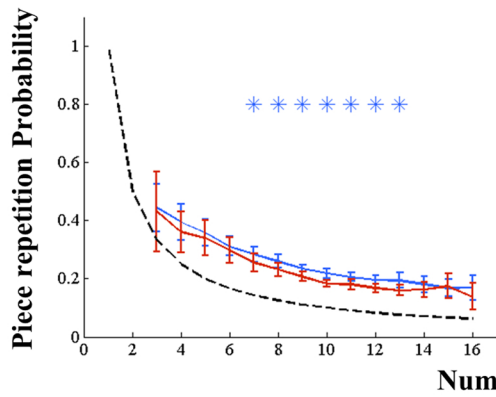**B****900 s**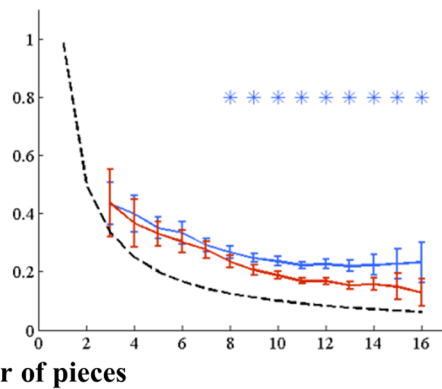**C**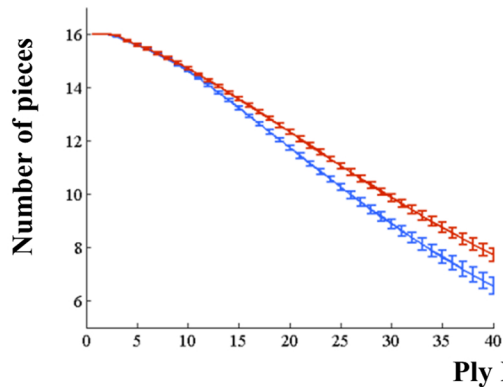**D**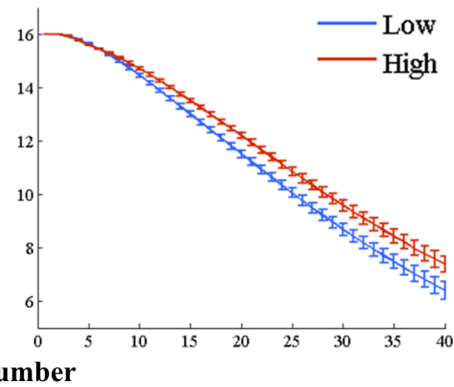

Supplement: Figure S3 — For longer games (300 and 900 s time budget), object-based mechanisms also depend on expertise level. (A,B) Weak players repeat the same piece on consecutive movements more frequently than strong players. As for 180 s games (Figure 2A), we plotted the probability to repeat the same piece on successive movements vs. the number of pieces for both expertise levels for 300 and 900 s games. To quantify these observations we first submitted the data to a Two-Way ANOVA test with number of pieces and expertise level as independent factors and their interactions. Results for 300 s games showed a main effect of both factors (Expertise, p < 0.0001, F = 41.9, df = 1; Number of Pieces: p < 0.0001, F = 61.6, df = 15 and Interaction, p < 0.0001, F = 10.1, df = 15). Results for 900 s games showed a main effect of both factors (Expertise, p < 0.0001, F = 197.8, df = 1; Number of Pieces: p < 0.0001, F = 89.7, df = 15 and Interaction, p < 0.0001, F = 31.4, df = 15). We followed these tests with independent two sample t-tests (corrected with a strict Bonferroni criterion for multiple comparisons) for each number of pieces left, comparing the distributions for high and low rated players. Each value of the distribution is obtained from one of the 35 different sets of each expertise level. All comparisons consistently showed greater repetition probability for lower than higher rated player. For 300 s games, this effect was significant for 7–14 pieces remaining on the board [t(34) < −4.9, p < 0.001]. For 900 s games, this effect was significant for 8–16 pieces remaining on the board [t(34) < −6.4, p < 0.001]. Dashed black line shows the random threshold. (C,D) Low-rated players reduce the number of pieces more rapidly than high-rated players. As it was previously showed for 180 s games, the number of remaining pieces over the board is significantly higher for high rated players almost for the whole game for both 300 and 900 s games. First, we made a two-way ANOVA test with number of pieces [file Presentation3.PDF]

5

10

20

30

40

Low

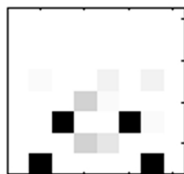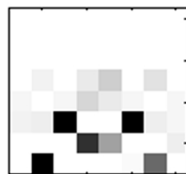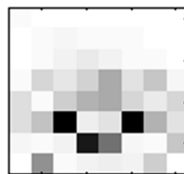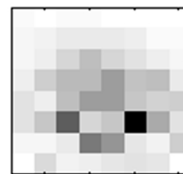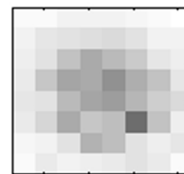

High

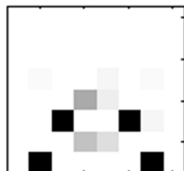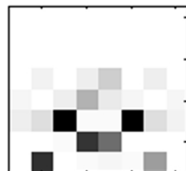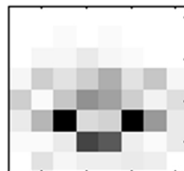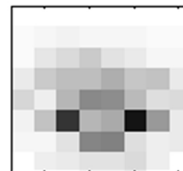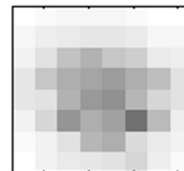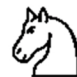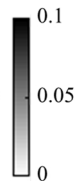

Low

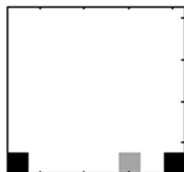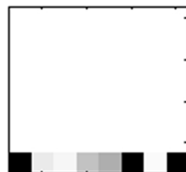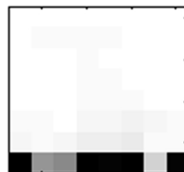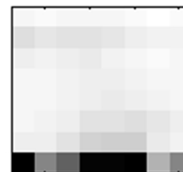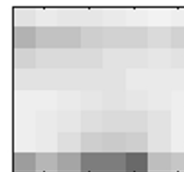

High

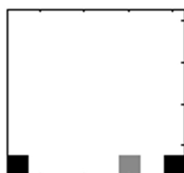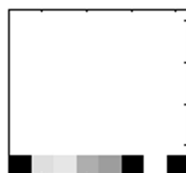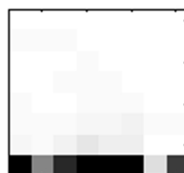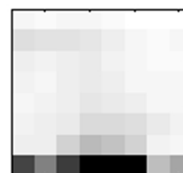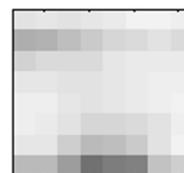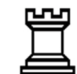

Low

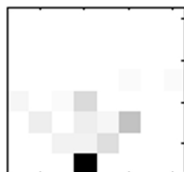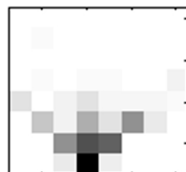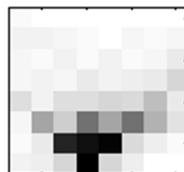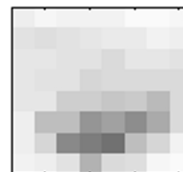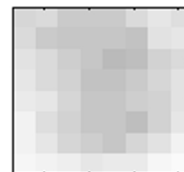

High

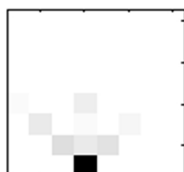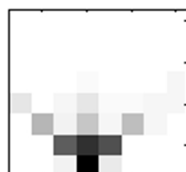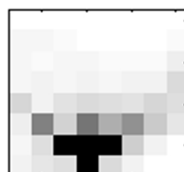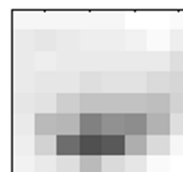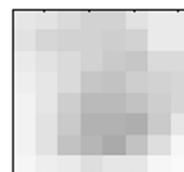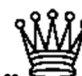

8  
7  
6  
5  
4  
3  
2  
1

Rows

a b c d e f g h  
Columns

Supplement: Figure S4 — Occupation distribution of pieces throughout the game for both expertise levels. Probabilities to find a (A) Knight, a (B) Rook, or the (C) Queen at each square of the chess board is represented for each rating group at different game stages (move numbers 5, 10, 20, 30, and 40). It should be noted that both groups occupy almost the same squares along the board (there are not “exclusive” squares), but some places are comparatively more occupied by weak or strong players (see Figure 3). [file Presentation4.PDF]

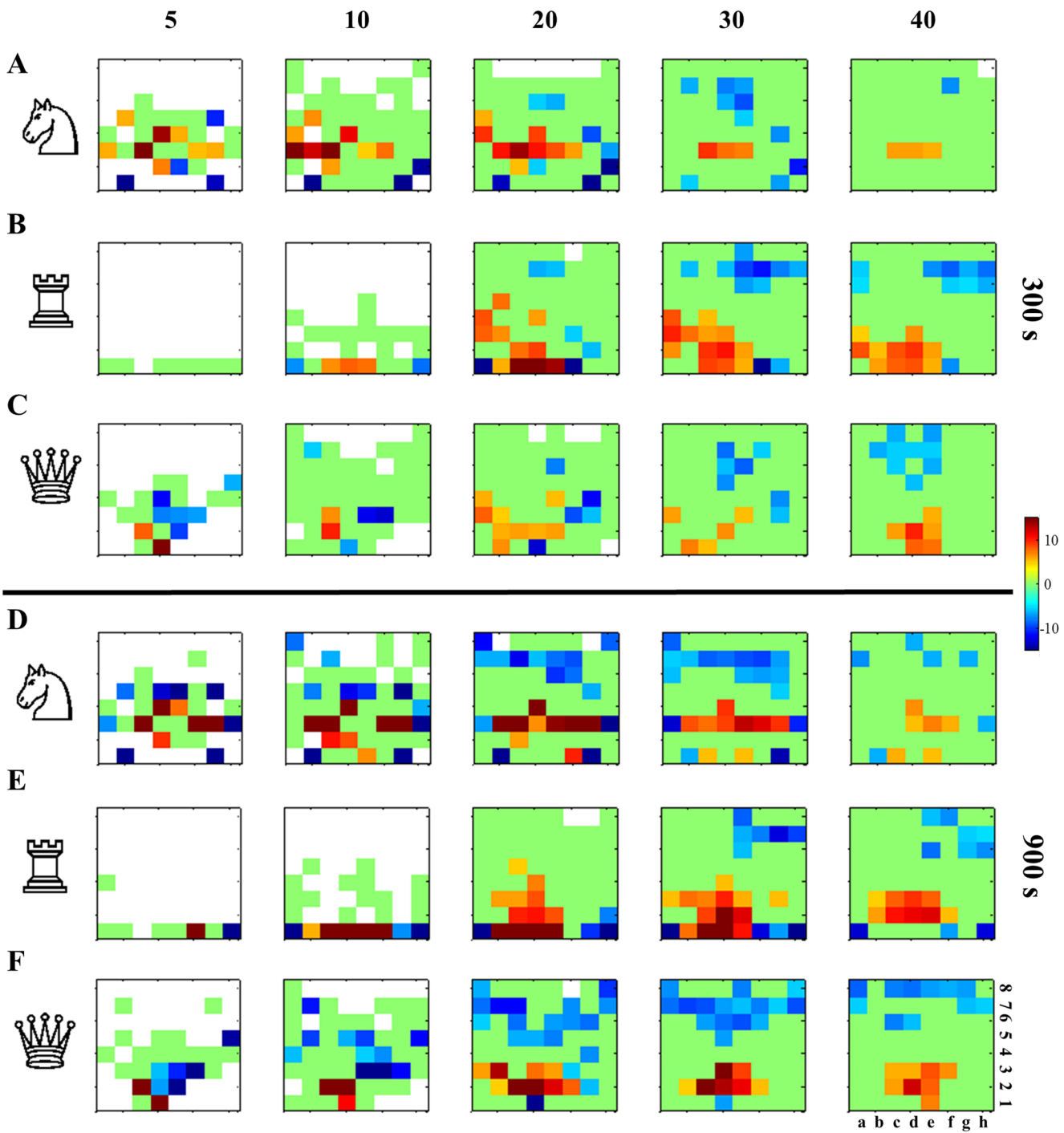

Supplement: Figure S5 — Piece distribution over the board also reveals domain-specific and expertise-dependent strategies in longer games (300 and 900 s time budget). Probabilities to find a type of piece (normalized by the number of remaining pieces of this type) in each square (8*8 checkerboard) were compared for high and low expertise groups at different game stages (moves 5, 10, 20, 30, and 40). The t-value resulting of the two-sample t-test (high vs. low expertise group), in each square is color coded for those significantly different comparisons with p < 0.001 Bonferroni corrected for multiple comparisons. Red positive values indicate significantly higher probabilities for strong players and blue negative values, significantly higher probabilities for weaker players. (A,D) Knights, (B,E) Rooks, and (C,F) Queen occupancy comparisons reveal that strong players centralize more their knights and rooks, delaying the queen development, compared with lower rated players delaying more the development of knights and rooks (but not the development of the queen, which is almost always not good) and/or occupying more advanced squares with all. It should be noted that the this figure represents the differential occupancy of each square, showing that strong or weak players locate each type of piece comparatively more frequently than the other group. (A–C) correspond to 300 s games. (D–F) correspond to 900 s games. [file Presentation5.PDF]
